# A Triboelectric-Based Artificial Whisker for Reactive Obstacle Avoidance and Local Mapping

**DOI:** 10.34133/2021/9864967

**Published:** 2021-07-10

**Authors:** Peng Xu, Xinyu Wang, Siyuan Wang, Tianyu Chen, Jianhua Liu, Jiaxi Zheng, Wenxiang Li, Minyi Xu, Jin Tao, Guangming Xie

**Affiliations:** ^1^ Marine Engineering College, Dalian Maritime University, Dalian 116026, China; ^2^ College of Artificial Intelligence, Nankai University, Tianjin 300350, China; ^3^ Department of Electrical Engineering and Automation, Aalto University, Espoo 02150, Finland; ^4^ Intelligent Biomimetic Design Lab, College of Engineering, Peking University, Beijing 100871, China; ^5^ Institute of Ocean Research, Peking University, Beijing 100871, China

## Abstract

Since designing efficient tactile sensors for autonomous robots is still a challenge, this paper proposes a perceptual system based on a bioinspired triboelectric whisker sensor (TWS) that is aimed at reactive obstacle avoidance and local mapping in unknown environments. The proposed TWS is based on a triboelectric nanogenerator (TENG) and mimics the structure of rat whisker follicles. It operates to generate an output voltage via triboelectrification and electrostatic induction between the PTFE pellet and copper films (0.3 mm thickness), where a forced whisker shaft displaces a PTFE pellet (10 mm diameter). With the help of a biologically inspired structural design, the artificial whisker sensor can sense the contact position and approximate the external stimulation area, particularly in a dark environment. To highlight this sensor’s applicability and scalability, we demonstrate different functions, such as controlling LED lights, reactive obstacle avoidance, and local mapping of autonomous surface vehicles. The results show that the proposed TWS can be used as a tactile sensor for reactive obstacle avoidance and local mapping in robotics.

## 1. Introduction

In recent years, much effort has been devoted to giving robots the capacity to assess their environment by equipping them with various sensors [1–3]. Typical sensors include gyroscopes [4], laser range finders [5], and inertial navigation systems [6]. Very few sensors used in robots provide tactile sensation [7, 8]. It is encouraging that nature has shaped effective biological tactile systems to sense complex stimuli generated by organisms’ motions. The tactile information received by animals is used for navigation and obstacle avoidance. Among these tactile sensing capabilities, animals’ whiskers have high sensitivity, specificity, and short response time, and such a tactile perception system could be mimicked in robots with the goal of recognizing the position and orientation of objects, particularly in a dark environment without any proper lighting. In earlier studies [9–11], models of animals’ whisker follicles involved very detailed representations of the mechanical and nerve structures, and these structures exhibit complex higher-order dynamics. The whisker’s bending moment was used to construct robotic whisker arrays that can extract precise information about object shape and fluid flow [12]. A piezoelectric material or Hall effect element could be used to design such a whisker [13] and distinguish two similar textures by sensing variations in stiffness [14]. In such a sensor array [15], the forces are transmitted along the vibrissae fibers to a load plate bonded to embedded MEMS barometers potted in polyurethane rubber, which act as force sensors. A fluid motion sensor was designed in previous studies [16–18], which was inspired by seals, who use their whiskers to find and follow underwater wakes. To date, triboelectric nanogenerators (TENGs) have not been built with a whisker-like structure.

To the best of our knowledge, few tactile sensors have been designed using various materials for use in industrial robots. Moreover, most of these sensors cannot work within the limited volume of robots and highly limited payload energy [19–21], which leads to high demand for sensors that are lightweight, small in size, are inexpensive, and have low power consumption. Besides developing new energy storage technologies, a self-powered whisker with high sensitivity could be useful for robot motion perception. Motivated by these problems, TENG coupled with triboelectrification and electrostatic induction has been developed as an electromechanical energy conversion technology; such a device exhibits potential applications in energy harvesting and self-powered mechanical sensing [22–28]. Self-powered triboelectric sensors have shown responses to environmental stimulation without requiring an energy source [29, 30], ultrahigh sensitivity to vocal intonations [31, 32], energy harvesting from water waves [24], acceleration perception [25], and human motion in real time [33–35]. A triboelectric wave device with a liquid-solid interface that uses various materials and has a simple structure was demonstrated in an earlier study [36–38]. These skin-like sensory devices allow them to naturally sense and interact with the surrounding environment [39–41]. With these capabilities, a triboelectric whisker-inspired sensor (TWS) may provide a simple solution, thereby providing robots with the ability to perceive motion.

**Figure 1:**
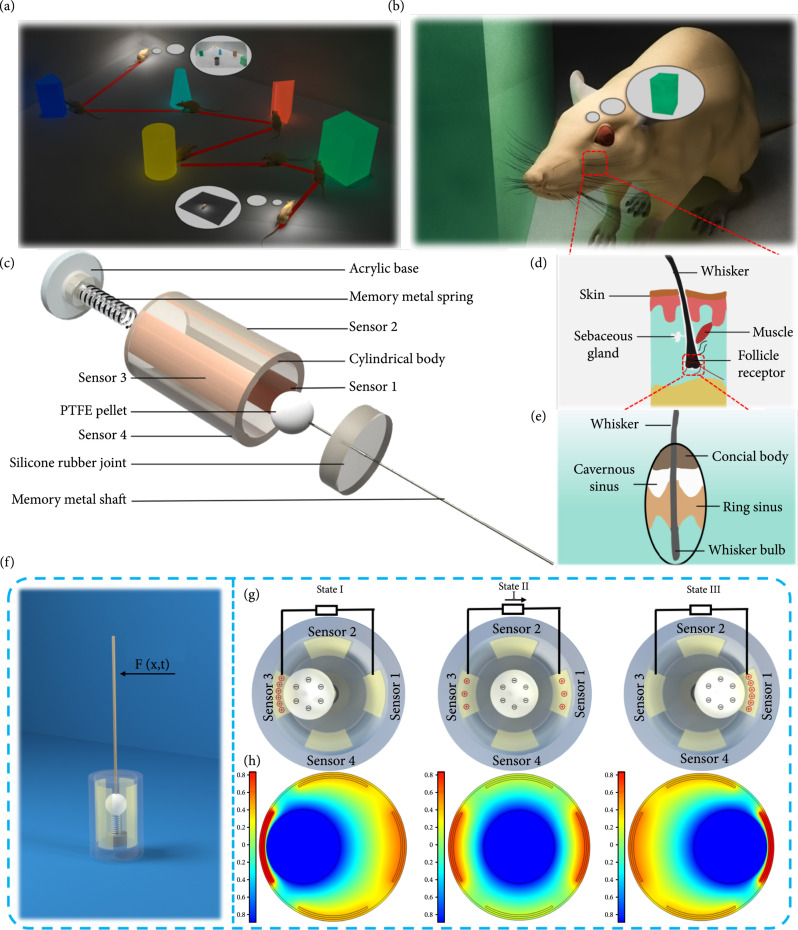
Structure and working mechanism of a TWS. (a) A mouse in darkness explores the environment with its whiskers. (b) Measuring both the orientations and distances from obstacles. (c) Location of tactile receptors beneath the surface of the skin. (d) The structure and innervation of a rat whisker follicle. (e) Basic structure of the bionic follicle whisker sensor. (f) Frontal view of the working components. (g) Schematic charge distribution as the PTFE pellet moves. (h) Simulation results showing the potential distribution between the PTFE pellet and Cu film.

Herein, we report an easy-fabrication, contact-separation channel TWS for use in robotic tactile perceptual systems, as shown in Figure 1. The TWS presented herein can be used to assess an unknown environment without requiring laser range finders or vision. Specially, the TWS can be used to measure the position and orientation of various objects by measuring the contact separation between the electropositive polytetrafluoroethylene (PTFE) pellet and the electronegative copper films. It is worth noting that the electrostatic effect mainly plays a major role in determining the output voltage when PTFE pellet and copper films are not in contact. Therefore, both the motion caused by tiny self-actuation and external stimuli can be perceived. Moreover, the directional load response from four sensors shows that this sensor can be used to distinguish load from directions. Finally, two cylindrical TWSs with four signal channels can be easily installed onto an autonomous surface vehicle (ASV), providing reactive obstacle avoidance and local mapping capabilities. In general, the proposed devices, together with test results from ASV, illustrate practical applications for the proposed sensors.

## 2. Results

### 2.1. Basic Structure and Working Mechanism of the TWS

A rat using its whiskers in darkness can run at high speeds without colliding with any surrounding obstacles, as shown in Figures 1(a) and 1(b). Figures 1(d) and 1(e) show the major components of a rat whisker follicle. These anatomical structures contain zones of innervation that are required to transduce mechanical signals from the whisker, as well as a mechanical structure that supports the whisker and influences its dynamic response to external perturbations. Based on this biological inspiration, the detailed structure of the TWS is designed for measuring both orientation and distance from an external load, as shown in Figure 1(c). The structure is composed of a soft silicone rubber joint (Ecoflex 00-20), a 3D-printed cylindrical housing (polylactic acid), and a rigid 3D-printed base. This base contains a spring that is attached to a PTFE pellet. A memory alloy shaft passes through the middle of a silicone rubber joint and connects to the PTFE pellet. Four copper films (0.3 mm) are attached symmetrically to the inner surface of the housing.

The starting position of the PTFE pellet is such that it can touch each Cu electrode, allowing any small deflection to produce a change in the output voltage. The sequence is as follows: any deflection in the whisker shaft forces the PTFE pellet to touch the Cu electrodes, and the sensor will produce an output voltage that is a quadratic function of deflection. Figure 1(g) shows an electricity generation cycle in a short-circuit condition. In Figure 1(f), when the PTFE pellet makes contact with the Cu film, opposite electric charges are induced on the surfaces of two materials via the triboelectric effect. The surface of the PTFE pellet becomes negatively charged, and the Cu film becomes positively charged because PTFE has higher electronegativity than Cu. When the PTFE pellet separates from the Cu film, a positive charge is generated on the opposite electrode as free electrons in the external circuit flow from the PTFE pellet to the Cu film and balance the electrical potential difference. When the whisker shaft touches an external obstacle from a different direction, an electric potential and current are generated again. Thereafter, the PTFE pellet moves back to the neutral position. Finally, the distribution of electric charge returns to its initial state, and an electrical signal generation cycle is completed. It is worth noting that the pellet cannot touch the copper electrode in order to ensure the TWS will produce a voltage via electrostatic induction. However, the value of the electrostatic signal is smaller than the output voltage produced by in contact and separation mode. The working principle of the TWS is further verified in Figure 1(h), which shows the simulated potential distribution between the PTFE pellet and Cu film in different positions; these results were obtained using finite element analysis in COMSOL. In this case, an alternating voltage is generated from the TWS.

### 2.2. Fundamental Characteristics of TWS

Figure S2(a) shows a schematic of the experimental setup used. For sake of clarity, the definition of 0° is given when the external load is applied along the 1 direction, as shown Figure S2(d). Similarly, the sensor’s directions are coded as 2: 90°, 3: 180°, and 4: 270°.

**Figure 2:**
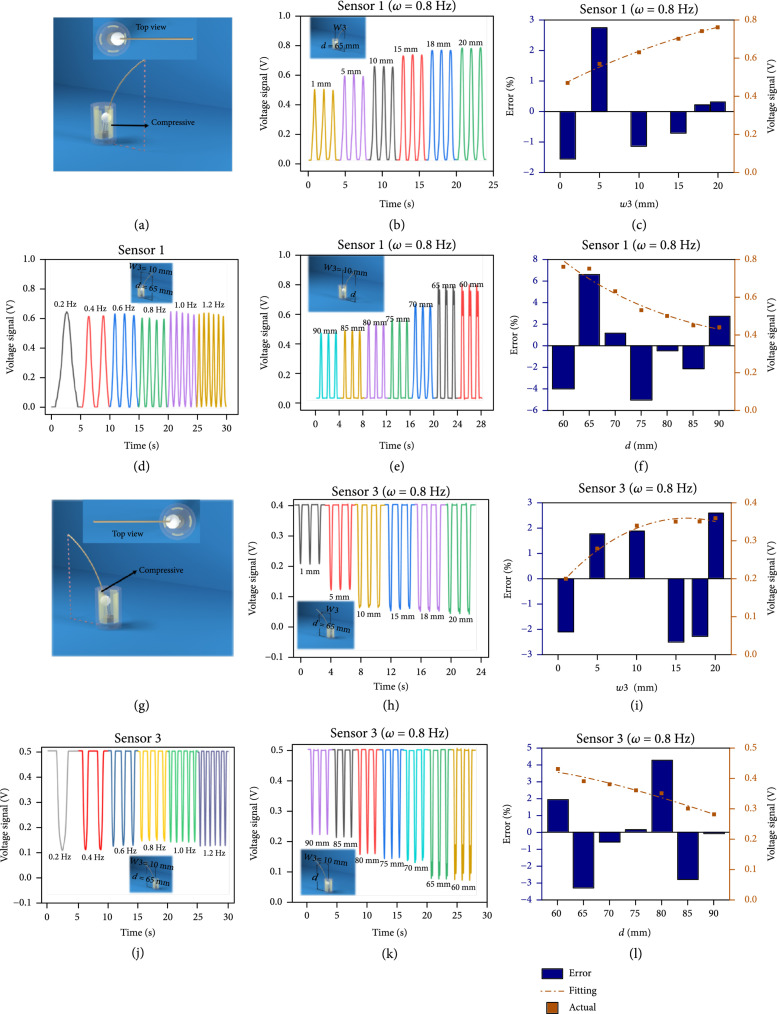
Experimental results. (a) The 3DMax model of the whisker sensor and its deflection along the 1 direction. (b) Response due to bending by $w_{3}=1\text{ mm}–20\text{ mm}$ in the 1 direction. (c) LOOCV validation for evaluating accuracy and generalization performance of 1 regarding $w_{3}$. (d) Response from 0.2 Hz to 1.2 Hz in the 1 direction. (e) Response at height d=60 mm–90 mm in the 1 direction. (f) LOOCV validation for evaluating accuracy and generalization performance of 1 regarding d. (g) 3DMax model of a whisker and deformation along the 3 direction from its relaxed state. (h) Response due to bending by $w_{3}=1\text{ mm}–20\text{ mm}$ along the 3 direction. (i) LOOCV validation for evaluating accuracy and generalization performance of 3 regarding $w_{3}$. (j) Response performance from 0.2 Hz to 1.2 Hz in the 3 direction. (k) Response performance at height d=60 mm–90 mm in the 3 direction. (l) LOOCV validation for evaluating accuracy and generalization performance of 3 regarding d.

Figure 2 shows data gathered along the 1 and 3 directions. A 3D model and the bent state of the TWS along the 1 direction are shown in Figure 2(a). When f=0.8 Hz and H=65 mm, Figure 2(b) shows the influence of the displacement $w_{3}$ on the output voltage from the TWS along the 1 direction. As the displacement $w_{3}$ gradually increases from 1 to 20 mm, the peak output voltage first increases and then reaches a plateau. This is because increasing the displacement $w_{3}$ can decrease the distance between the PTFE pellet and Cu electrodes and increase their contact forces. From [22], increasing mechanical compression between the PTFE pellet and Cu electrodes causes an increased output voltage. However, due to material limitations and the size of the TWS, the output voltage saturates the sensor. Moreover, a leave-one-out cross-validation (LOOCV) strategy was used to fit the 1 model, and the detection accuracy and generalization performance of these models are shown in Figure 2(c). This confirms that the quadratic model has a high correlation coefficient of 0.99382 at all displacement values, with errors being less than 0.22% at displacement $w_{3}=18\text{ mm}$, and a relatively higher error of 2.7% at displacement $w_{3}=5\text{ mm}$. These errors may be caused by environmental vibration from the motor. Figure 2(d) shows the influence of the collision frequencies when H=65 mm and $w_{3}=15\text{ mm}$ along the 1 direction. Evidently, the output voltage of the 1 direction is nearly constant when the frequency increases from 0.2 to 1.2 Hz. Frequency has little effect on the voltage output from the TWS. Figure 2(e) describes the influence of the collision height on the output voltage from the TWS along the 1 direction when $w_{3}=18\text{ mm}$ and f=0.8 Hz. Contrary to the case where $w_{3}$ increases, the peak output voltage first decreases and then plateaus as the collision height gradually increase from 60 to 90 mm. This is because the displacement of the PEFT pellet along the 1 direction decreases as the collision height gradually increases. Using the LOOCV strategy, the detection accuracy and generalization performance of 1 model regarding the collision height are shown in Figure 2(f). The output voltage also fits well to a quadratic function of collision height with a high correlation coefficient of 0.96027. Together with 1 model regarding the displacement as shown in Figure 2(c), the quadratic models describing the TWS and simulating shaft motion suggest that the detection accuracy is sufficient to use the whisker in robotic applications. The data along the 3 direction are plotted in Figures 2(g)–2(l). Note that, in contrast to data from the 1 direction, the falling edge is generated at peak output voltage values, as shown in Figures 2(h), 2(j), and 2(k). Therefore, the deflection direction can be determined using the output voltage. Similar data along the 2 and 4 directions are plotted in Figure S3. We observed that for all directions, the collisions associated with data sets correlate them with object distances. In addition, a silicone rubber joint is designed to provide damping and support the device. Table S1 and Figure S1 show how 3 silicone samples with different hardness affect the output voltage. One can see that the sensor is greatly disturbed by vibration when no silicone rubber is used. However, when a harder silicone is used for damping, the output value caused by environmental vibration will decrease. This means that the design of using silicone rubber provides TWS with more stable performance in practical applications. On the other hand, because the silicone rubber and spring can help support the whisker shaft when a TWS is placed horizontally, we can ignore the influence of gravity when the whisker shaft is forced by some external stimulus. As shown in Figure S2, there is almost no difference between the output voltage placed vertically and horizontally. The results show that the output voltage will be the same regardless of orientation. Consequently, a perceptual system based on TWS reveals technical abilities to navigation, local mapping, and object recognition.

### 2.3. Real-Time Control Verification

**Figure 3:**
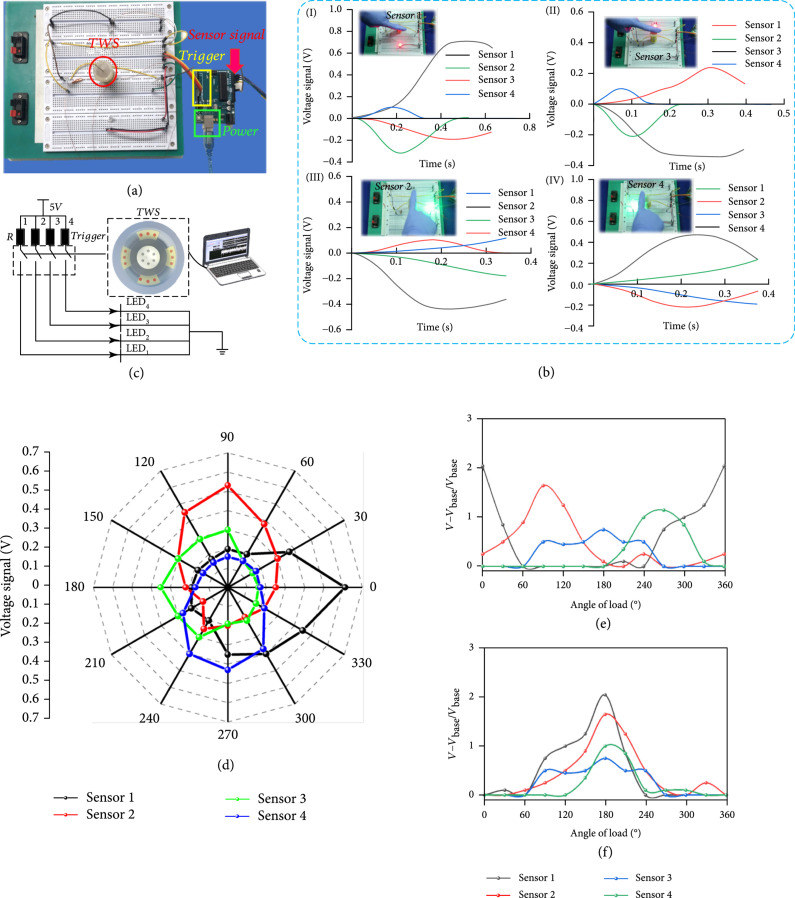
Experimental results. (a) Experimental electronic setup. (b) Demonstration of TWS as a sensitive load switch control and its corresponding output voltage signal. (c) Electronic module used for potential application demonstrations, such as controlling LED lights. (d) Directional patterns of the TWS. (e) Rotation from 0° to 360 and ΔV for each angle with the same load applied. (f) The results of (e) were replotted with 0 defined.

Figure 3(a) shows a photograph of the experimental electronic setup with a signal sampling module and a trigger signal transform module. The corresponding circuit is depicted in Figure 3(c). Upon application of a load stimulus to the TWS, a triboelectric output voltage is generated. The electrical output from the TWS was used to generate a stimulating pulse for turning on LED lights in a corresponding direction. The LED light module responds to the corresponding control signals from a trigger signal transform module. Figure 3(b) shows how the TWS is used to control the on/off state of LED lights (movie S1) by a corresponding voltage. The experimental results indicate that (i) the load stimuli direction produces a larger voltage compared with other directions, and (ii) a higher load stimulus frequency produces an AC voltage with a larger frequency.

An experiment was conducted out with a load being applied to the side of the whisker shaft at every 30°. The directional load response from four sensors when f=0.8 Hz, H=65 mm, and $w_{3}=15$ is shown in Figure 3(d). An asymmetric pattern can be seen in the figure, which results from errors in the production process. From Figure 3(d), the sensors 1 and 2 exhibit a higher voltage signal under the same experimental conditions, which implies the position of the PTFE pellet is closer to 1 and 2.

Generally, when the deviation angle for every sensor increases, the peak output voltage gradually decreases. This is because the contact area decreases as the deviation angle increases for every sensor. For example, once the deviation angle for sensor 1 is greater than 60°, all voltage outputs fall into a circle of 0.2 V shown in Figure 3(d), which stems from electrostatic induction. Namely, the PTFE pellet cannot make contact with the Cu electrode in sensor 1 in this case. Here, $V_{\text{base}}=0.2\text{ V}$ is regarded as a base voltage. The relationship between the normalized voltage change $\Delta V=V-V_{\text{base}}/V_{\text{base}}$ as a function of time, which measures the response time of the whisker sensor, is plotted in Figure 3(e). The trend for each sensor is similar to a sine curve. To gain a better understanding about the ΔV trend for each sensor, the results in Figure 3(e) were replotted and presented, as shown in Figure 3(f), where the relative position of each sensor is denoted as 0°. The four sensors appear to produce consistent results, which verify the shaft is elastically balanced as we expected. From the shaft position and the load direction data in Figure 3(f), one can see that the ratio ΔV changes from 0° and ±60° at the lowest point and from 120° to 240° at the highest point, which suggests that the fabricated whisker sensor is mechanically flexible and stable. The experimental results reveal the effectiveness of the proposed assembly process, i.e., the center shaft can be elastically balanced.

### 2.4. Applications of the TWS in Reactive Obstacle Avoidance

To further demonstrate the effectiveness and the applicability of the designed TWS, we present some experimental results with a JetBot robot. JetBot is an open-source robot built from the NVIDIA Jetson Nano platform, which is suitable for investigating the ideas proposed in this paper.

**Figure 4:**
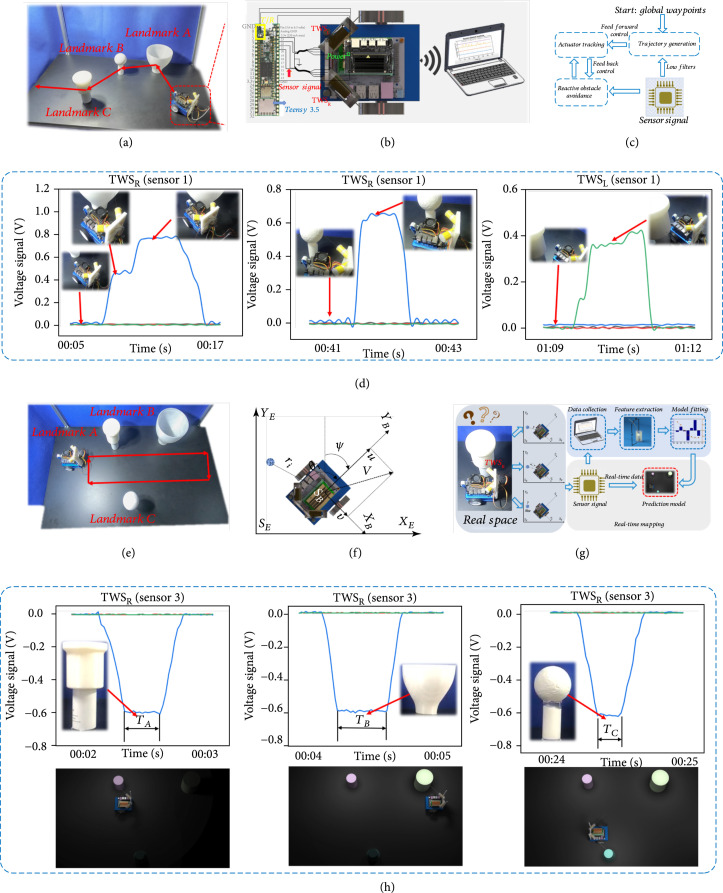
Experimental results. (a) Photographs of the actual workspace for reactive obstacle avoidance. (b) Electronic module used for potential application demonstrations, such as reactive obstacle avoidance and local mapping. (c) Overview of the closed-loop control system for reactive obstacle avoidance. (d) Voltage signal measured at landmarks A, B, and C. (e) Photographs of the actual workspace used for local mapping. (f) Reference frames: BODY reference frame and NED reference frame. (g) Local mapping process, where sensory information is applied for model fitting and real-time model prediction. (h) Voltage signal measured at landmarks A, B, and C.

The workspace consists of a 3.66 m by 3.66 m enclosed region, and three stationary landmarks are placed in this region, as shown in Figure 4(a). With a small and simple structure, two cylindrical TWSs with four signal channels can be easily installed on the JetBot to detect the position and orientation of each landmark. Figure 4(b) shows that information from the TWSs can be sampled and then transmitted to the JetBot using a Teensy 3.5 module. An ARM A57 processor was used for data processing, which provides 472 GFLOPS (billion floating-point operations per second) with only 5 W of power consumption. Information gathered by the JetBot is sent to a computer and vice versa through a Wi-Fi network. In addition, to better track the trajectory between position updates, the JetBot utilizes dead reckoning from encoder information located on the drive motors.

In the prescribed workspace, a preplanned path is traversed by a cascade controller, where feedforward and feedback controls are used to ensure high accuracy, in which JetBot is fully autonomous as it drives along the preplanned path. It is worth noting that the robot adopts a stepping movement with maximum speed of 1 cm/s as JetBot has enough time to respond to the TWS, while a robot with higher speed could cross the workspace boundary in the limited experiential space. Figure 4(c) shows the process used to implement reactive obstacle avoidance. As soon as a landmark is recognised by the TWS, the feedback controller determines the direction of deviation from the obstacle, and the robot deviates from its original trajectory. Moreover, JetBot first moves in reverse to generate the a maximum longitudinal displacement of 5 cm, and then, it turns 15° in the determined direction. JetBot then returns to the original trajectory as soon as it has cleared a path away from the landmark. Figure 4(d) shows the corresponding voltage signal (see Supplementary Movie S2), when the TWS is in contact with landmarks A, B, and C. This demonstration shows the feasibility of using the TWS for constructing an electronic reactive obstacle avoidance system with low-cost and accurate tactile sensing.

### 2.5. Applications of the TWS in Local Mapping

Similar to testing reactive obstacle avoidance, three stationary landmarks are relocated in the test workspace, as shown in Figure 4(e). The vehicle starts at the origin and remains stationary for approximately 2 s; then, it executes a series of loops at speeds up to 2 cm/s. The preplanned vehicle path is shown with a red solid line in Figure 4(e). The process for establishing and using local mapping is shown in Figure 4(g). Real-time recognition and mapping is achieved using an antifitting method for model fitting based on the fitted quadratic model (also see Supplementary Movie S3), where the screen displays both the real-time signals and the corresponding collision objects, as shown in Figure 4(h). The signal bandwidth depends upon the size of the landmarks. Consequently, we can roughly estimate the size of the landmarks using local mapping. The prototype of TWS-based mapping method shows potential for creating a new type of simultaneous localization and mapping in tactile applications.

## 3. Discussion

A tactile perception system with two integrated TWSs is proposed for adding reactive obstacle avoidance and local mapping capabilities to robots. The sensor can be used to map the contact area of external stimuli by using the triboelectric and electrostatic output from a Cu film and PTFE pellet. This is because animal whisker models exhibit complex higher-order dynamics [9–11], providing an accurate position and orientation determination for various objects. The use of a memory alloy spring ensures measurements are reproducible. In contrast with earlier studies [15–17], this sensor also exhibits a directional load response and high sensitivity to external stimuli, due to the no-contact electrostatic or contact-electrification principle of TENG [22]. Moreover, using the signal data, model fitting with respect to $w_{3}$ and d is carried out. Two cylindrical TWSs were installed on the JetBot to detect landmark positions and their orientation using a regression model, providing reactive obstacle avoidance and local mapping capabilities. In this tactile perception system, signals are processed in real time, and the system can be used in automation applications.

## 4. Methods

### 4.1. Fabrication of the TWS

The soft silicone rubber joint was made of Ecoflex 00-20 silica gel. To be specific, 10 ml of solution A and 10 ml of solution B of Ecoflex 00-20 silica gel were mixed in a petri dish with a 1 : 1 volume ratio and stirred with an electric stirrer at 120 rpm for 2-3 min. Then, a vacuum pump was used to vacuum the mixture to -0.1 MP for 2 min. The vacuumed silica gel mixture was poured into the 3D-printed mold and cooled at 25° for 36 h. This process yields a silicone rubber joint of X mm diameter. A cylindrical housing (size) and a rigid base (size) were 3D-printed. On the top of the rigid base center, there was a 10 mm deep screw thread for connecting the bottom of the spring, and the spring’s top was fixed to a 10 mm diameter PTFE pellet by EVA hot melt adhesive. A small hole with a 5 mm depth was placed on top of the PTFE pellet so that a 50 mm long Ti-Ni memory alloy wire could be placed in the pellet. EVA hot melt adhesive was used to bond the Ti-Ni memory alloy wire to the PTFE pellet. The Ti-Ni memory alloy wire passed through the silicone rubber joint. A 0.3 mm thick copper film was cut into 10∗30 mm^2^ to form electrodes, and these sections were symmetrically distributed on the housing’s inner wall with adhesive. The induced voltage was transmitted by 0.3 mm thick copper wires attached to copper electrodes.

### 4.2. Characterization and Electrical Measurement

The TWS is vertically attached to a fixture adapter that is mounted on a linear motor (LINMOT EI200-P01). At different frequencies and amplitudes, a linear motor can be used to drive the TWS so that it made contact with obstacles. The signal produced by the TWS was measured with a Keithley 6514 electrometer, and the measurement was displayed in a custom Labview VI. A photo of the experimental setup is shown in Figure S2(b) and (c). To determine the highest sensitivity of the TWS, triboelectrification and electrostatic induction data were collected separately by bending the whisker shaft along four directions.

## Data Availability

The authors declare that the main data supporting the findings of this study are available within the article and its Supporting Information files. Extra data are available from the corresponding authors on reasonable request.

## Supplementary Materials

Supplementary 1 Table S1: three silicone joint for experimental study. Figure S1: TWS dynamic model. Figure S2: (a) Schematic of the experimental setup. (b) Hardware setup. (c) TWS being used to probe an obstacle. (d) Definition of the angle at which the load was applied. Figure S3: experimental results. (a) 3DMax model of a whisker and its deformation along the 2 direction starting from a relaxed state. (b) Response by bending to $w_{3}=1‐20$ mm along the 2 direction. (c) LOOCV validation for evaluating accuracy and generalization ability of 2 regarding $w_{3}$. (d) Response from 0 : 2 to 1 : 2 Hz in the 2 direction. (e) Response performance at height d=60 mm‐90 mm in the 2 direction. (f) LOOCV validation for evaluating accuracy and generalization of 2 regarding d. (g) 3DMax model of whisker and deformation representation along the 4 direction from relaxed state. (h) Response performance by bending $w_{3}=1‐20$ mm along the 4 direction. (i) LOOCV validation for evaluating accuracy and generalization of 4 regarding $w_{3}$. (j) Response from 0 : 2 to 1 : 2 Hz along the 4 direction. (k) Response at height d=60–90 mm along the 4 direction. (l) LOOCV validation for evaluating accuracy and generalization ability of 4 regarding d.
