# Supplementary material for: A Triboelectric-Based Artificial Whisker for Reactive Obstacle Avoidance and Local Mapping
Source: Research (Wash D C). 2021 Jul 10;2021:9864967. doi: 10.34133/2021/9864967 (PMC11014677; doi:10.34133/2021/9864967)
Supplement: Supplementary 1 — Table S1: three silicone joint for experimental study. Figure S1: TWS dynamic model. Figure S2: (a) Schematic of the experimental setup. (b) Hardware setup. (c) TWS being used to probe an obstacle. (d) Definition of the angle at which the load was applied. Figure S3: experimental results. (a) 3DMax model of a whisker and its deformation along the 2 direction starting from a relaxed state. (b) Response by bending to w3=1‐20 mm along the 2 direction. (c) LOOCV validation for evaluating accuracy and generalization ability of 2 regarding w3. (d) Response from 0 : 2 to 1 : 2 Hz in the 2 direction. (e) Response performance at height d=60 mm‐90 mm in the 2 direction. (f) LOOCV validation for evaluating accuracy and generalization of 2 regarding d. (g) 3DMax model of whisker and deformation representation along the 4 direction from relaxed state. (h) Response performance by bending w3=1‐20 mm along the 4 direction. (i) LOOCV validation for evaluating accuracy and generalization of 4 regarding w3. (j) Response from 0 : 2 to 1 : 2 Hz along the 4 direction. (k) Response at height d=60–90 mm along the 4 direction. (l) LOOCV validation for evaluating accuracy and generalization ability of 4 regarding d. [file 9864967.f1.zip › 9864967.f1.pptx]

## Slide 1
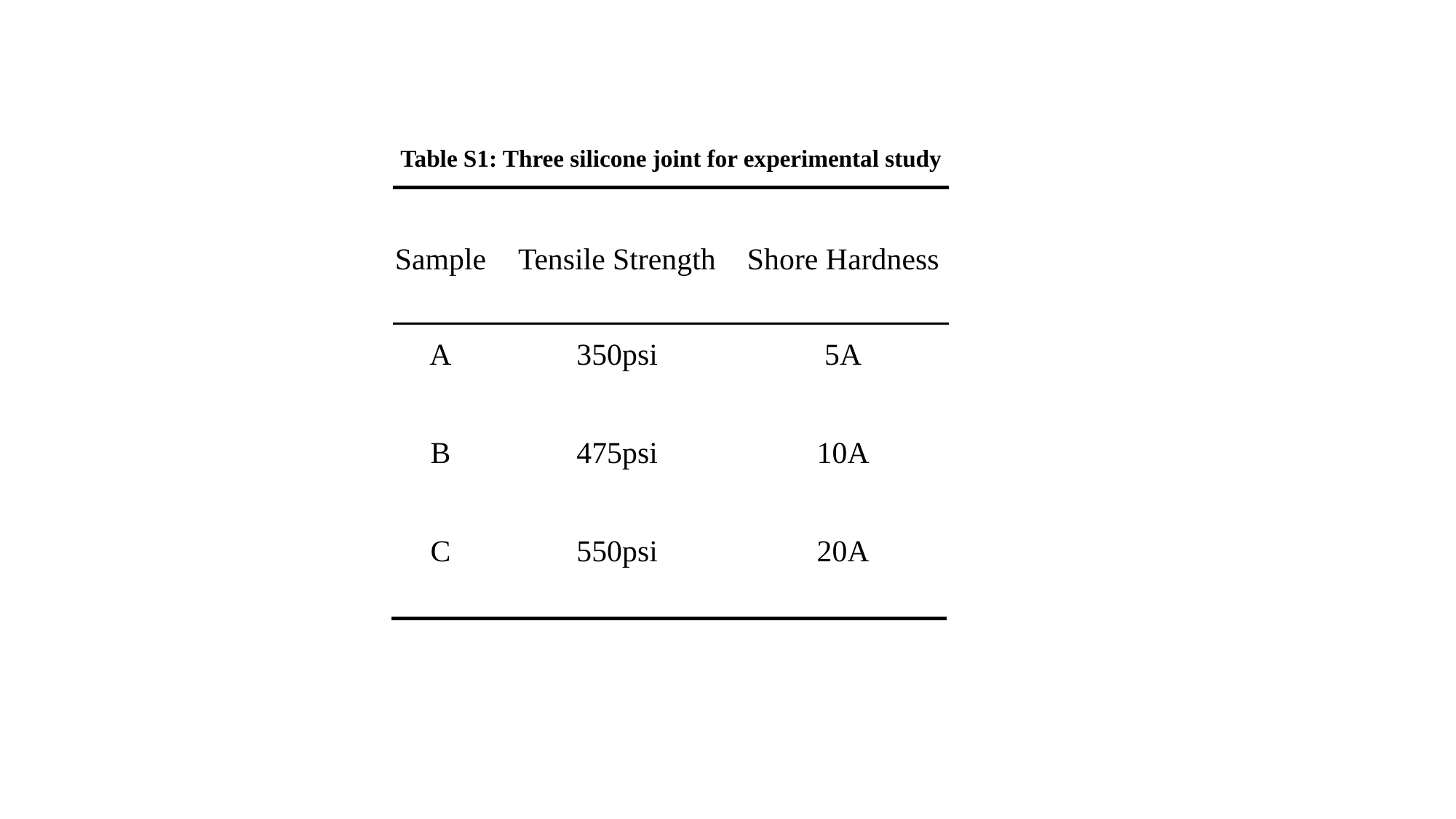

Table S1: Three silicone joint for experimental study
| Sample | Tensile Strength | Shore Hardness |
| --- | --- | --- |
| A | 350psi | 5A |
| B | 475psi | 10A |
| C | 550psi | 20A |
