# Supplementary material for: A Triboelectric-Based Artificial Whisker for Reactive Obstacle Avoidance and Local Mapping
Source: Research (Wash D C). 2021 Jul 10;2021:9864967. doi: 10.34133/2021/9864967 (PMC11014677; doi:10.34133/2021/9864967)
Supplement: Supplementary 1 — Table S1: three silicone joint for experimental study. Figure S1: TWS dynamic model. Figure S2: (a) Schematic of the experimental setup. (b) Hardware setup. (c) TWS being used to probe an obstacle. (d) Definition of the angle at which the load was applied. Figure S3: experimental results. (a) 3DMax model of a whisker and its deformation along the 2 direction starting from a relaxed state. (b) Response by bending to w3=1‐20 mm along the 2 direction. (c) LOOCV validation for evaluating accuracy and generalization ability of 2 regarding w3. (d) Response from 0 : 2 to 1 : 2 Hz in the 2 direction. (e) Response performance at height d=60 mm‐90 mm in the 2 direction. (f) LOOCV validation for evaluating accuracy and generalization of 2 regarding d. (g) 3DMax model of whisker and deformation representation along the 4 direction from relaxed state. (h) Response performance by bending w3=1‐20 mm along the 4 direction. (i) LOOCV validation for evaluating accuracy and generalization of 4 regarding w3. (j) Response from 0 : 2 to 1 : 2 Hz along the 4 direction. (k) Response at height d=60–90 mm along the 4 direction. (l) LOOCV validation for evaluating accuracy and generalization ability of 4 regarding d. [file 9864967.f1.zip › 9864967.f2.pdf]

A Triboelectric-Based Artificial Whisker for Reactive Obstacle Avoidance and Local Mapping

Peng Xu[a,*], Xingyu Wang[a,*], Siyuan Wang[a], Tianyu Chen[a], Jianhua Liu[a], Jiaxi Zheng[a], Wenxiang Li[a], Minyi Xu[a,**], Jin Tao[d,e,**], Guangming Xie[b,c,**]

[a]*Marine Engineering College, Dalian Maritime University, Dalian 116026, China*
[b]*Intelligent Biomimetic Design Lab, College of Engineering, Peking University, Beijing 100871, China*
[c]*Institute of Ocean Research, Peking University, Beijing 100871, China*
[d]*College of Artifical Intelligence, Nankai University, Tianjin 300350, China*
[e]*Department of Electrical Engineering and Automation, Aalto University, Espoo 02150, Finland*

Keywords: Triboelectric nanogenerator; Artificial whisker; Tactile sensor; Whisker follicles; Local mapping; Obstacle avoidance

1. Supporting Figures

2. Supporting Notes

2.1. Note S1. TWS Modal Analysis

The vibration dynamics of the TWS are described using a simple Euler-Bernoulli undamped beam model with uniform cross-section, as shown Fig S1(a) and (b). It is worth noting that, compared to the beam shear strain, the axial strain and cross-sectional rotation can be neglected. The model is justifiable since, on the one hand, rat whiskers exert miniscule contact forces, yet movements of a rat's whiskers are very rapid. The relationship between the external contact forces and the whisker deformation is [35]:

$$EI\partial^4 w/\partial x^4 + \rho A\partial^2 w/\partial t^2 = F(x,t), \tag{1}$$

*These authors contributed equally
**Corresponding author
Email addresses: xuminyi@dlmu.edu.cn (Minyi Xu), taoj@nankai.edu.cn (Jin Tao), xiegming@pku.edu.cn (Guangming Xie)

where w denotes the beam lateral displacement, x is the beam's axial location, and $F(x,t)$ is the external load at time t. $E = 28GPa$ and $I = \frac{\pi r^4}{4}$ are the beam elasticity and second moment of area, respectively. A, $\rho = 6400Kg/m^3$ and $r = 0.5mm$ are the beam cross-sectional area, density of the memory alloy shaft material, and radius, respectively.

Moreover, the boundary conditions with respect to $k_1 = 0.28N/mm$ are equal to the base linear springs, and $k_2 = 0.28N/mm$ and $k_\theta = 200Nm/rad$ are equal to the soft silicone rubber joint. The local shear force and moment at $x = 0$ are expressed using load balance equations as follows:

$$\begin{aligned}
&EI\partial^3 w(d_1)/\partial x^3 + k_1 w(d_1) = 0,\\
&EI\partial^2 w(d_2)/\partial x^2 - k_\theta \partial w(d_2)/\partial x = 0,\\
&EI\partial^3 w(d_2)/\partial x^3 + k_2 w(d_2) = 0,\\
&\partial w(0)/\partial x = w(0) = 0,\\
&\partial^3 w(d_3)/\partial x^3 = \partial^2 w(d_3)/\partial x^2 = 0.
\end{aligned} \tag{2}$$

Note that the moment balance is goverened by a 2nd-order differential equation. The natural frequencies of the beam can be calculated via separation of variables with $w(x,t) = W(x)T(t)$. Moreover, (1) can be rewritten as with negligible $F(x,t)$

$$EId^4W/dx^4 - \rho A\omega^2 W = 0. \tag{3}$$

This boundary value problem was solved using the Matlab bvp5c function. The simulation results in Fig. S1(c) show the lateral displacement response $W(x)$ and its derivative for first natural frequency $\omega_n \approx 5.81Hz$. When establishing a mechanical model, we assume the following: 1. The whisker shaft experiences small deformation; 2. The whisker shaft is stressed for a very short period of time. Compared with low-order natural frequencies, generating higher-order natural frequencies requires more energy consumption, which is opposite to prior model assumptions. Thus, Fig. S1(d) only shows the mode shapes for the first three natural frequencies $\omega_n \approx [5.81, 36.43, 102.02]Hz$. These results show that the correlation between sen-

sor readings may significantly change depending on the dominant natural frequency of vibration. Fig. S1(e) and (f) show the beam mode shapes for six different stiffness coefficients k_1. The stiffness significantly affects the beam mode shape for a given natural frequency. This means that the TWS sensitivity can be changed by controlling the stiffness of the whisker follicle.

2.2. Note S2. Local Mapping Strategy

Fig. 4(f) shows that two reference frames, a BODY reference frame S_B and an NED reference frame S_E, are often used to describe position and velocity in the horizontal plane. For the JetBot robot, let $\eta = [x, y, \psi] \in \mathbb{R}^3$ be the position (i.e., x, y) and orientation (i.e., ψ) in S_E, and $\nu = [u, v, r] \in \mathbb{R}^3$ be the linear velocity (i.e., u, v) and angular velocity (i.e., r) in S_B. Usually, the motion of JetBot can be described by using a nonlinear discrete-time model of three degrees of freedom using kinematics and kinetics:

$$\eta(k+1) = \eta(k) + R(\psi)\Delta T\nu(k), \tag{4}$$

where

$$R(\psi) = \begin{bmatrix} cos(\psi) & -sin(\psi) & 0 \\ sin(\psi) & cos(\psi) & 0 \\ 0 & 0 & 1 \end{bmatrix}. \tag{5}$$

Here, (4) can be used in the prediction stage of the vehicle state estimator. The landmarks in the environment are assumed to be stationary point targets. The landmark process model in S_E is thus:

$$\begin{bmatrix} L_x^i(k+1) \\ L_y^i(k+1) \end{bmatrix} = \begin{bmatrix} L_x^i(k) \\ L_y^i(k) \end{bmatrix} \tag{6}$$

for all landmarks $i = 1, 2, and 3$. The augmented state transition model for the complete system may now be written as follows:

$$\begin{bmatrix} \eta(k+1) \\ L_x^i(k+1) \\ L_y^i(k+1) \end{bmatrix} = \begin{bmatrix} R(\psi)\Delta T\nu(k) \\ L_x^i(k) \\ L_y^i(k) \end{bmatrix} + \begin{bmatrix} \nu_\eta(k) \\ 0 \\ 0 \end{bmatrix}, \tag{7}$$

where $\nu_\eta(k) = [\nu_x(k), \nu_y(k), \nu_\psi(k)]$ denotes temporally uncorrelated process noise. The TWS used in the experiments returns the range $r_i(k)$ and bearing $\theta_i(k)$ with respect to a landmark. Referring to Fig. 4(f), the observation model can be written as

$$
\begin{aligned}
r_i(k) &= \sqrt{(x_i - x(k))^2 + (y_i - y(k))^2} + \nu_r(k) \\
\theta_i(k) &= acrtan\left(\frac{y_i - y(k)}{x_i - x(k)}\right) - \psi(k) + \nu_\theta(k),
\end{aligned}
\tag{8}
$$

where $w(k)$ and $w_\theta(k)$ are the noise sequences associated with the range and bearing measurements. Subsequently, an extended Kalman filter (EKF) is used to generate estimates. The EKF uses linearized kinematic and observation equations for generating state predictions. The local mapping algorithm is described in detail in Appendix 2 of [36].

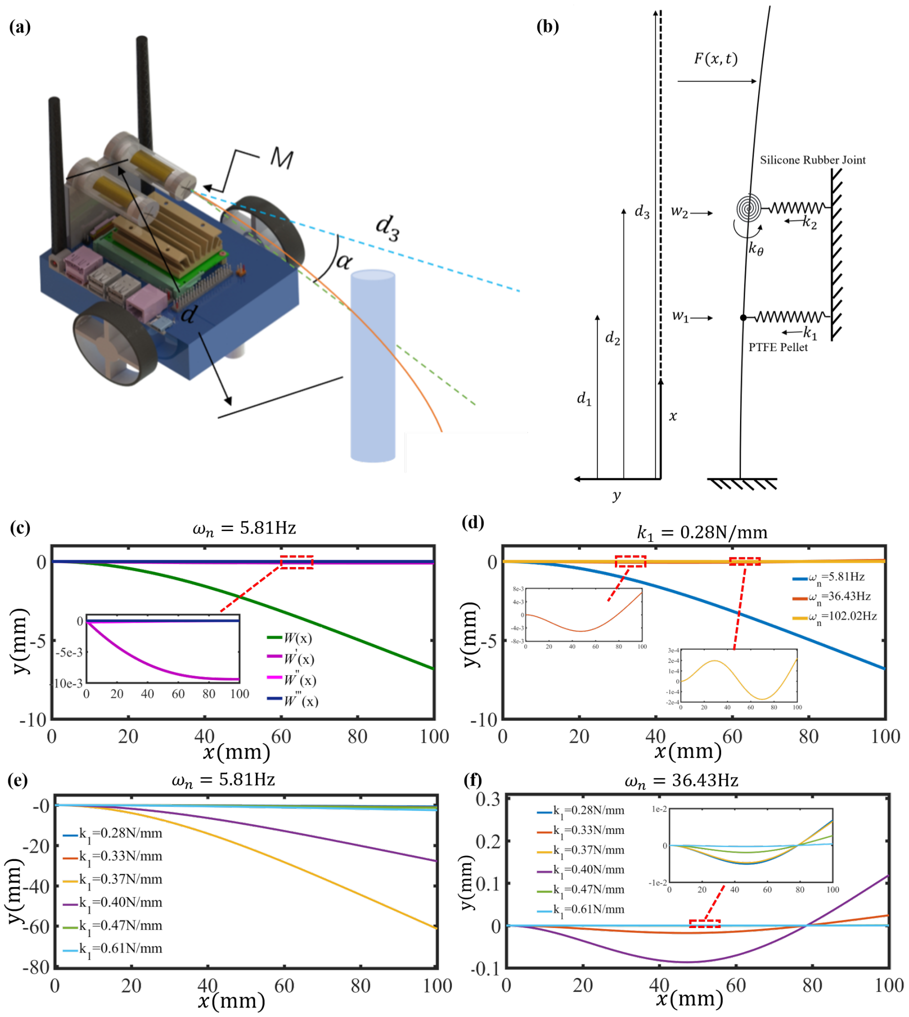

Figure S1: TWS dynamic model. (a) A shaft of length $d_3 = 70mm$ rotates through an angle α against an object a distance d away, which generates a moment M at the base. (b) A simple lumped parameter model describing the vibration dynamics of the TWS. The silicone joint is described by a torsion spring (k_θ) and a linear spring k_2 at $d_2 = 50mm$. The constraint of the PTFE pellet is represented by a linear spring k_1 at $d_1 = 30mm$. w_1 and w_2 are the beam lateral displacements at PTFE pellet and the silicone joint, respectively. (c) Simulated lateral displacement response $W(x)$ and its derivative for the first natural frequency. (d) Simulated mode shapes versus follicle stiffness values for three natural frequencies. (e) Simulated mode shapes versus different follicle stiffness values for the first natural frequency. (f) Simulated mode shapes against follicle stiffness values for the second natural frequency.

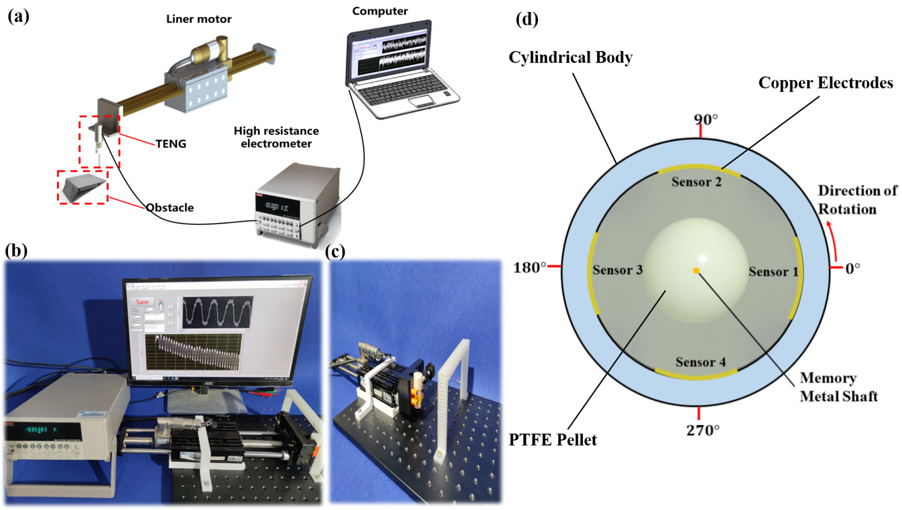

Figure S2: (a) Schematic of the experimental setup. (b) Hardware setup. (c) TWS being used to probe an obstacle. (d) Definition of the angle at which the load was applied.

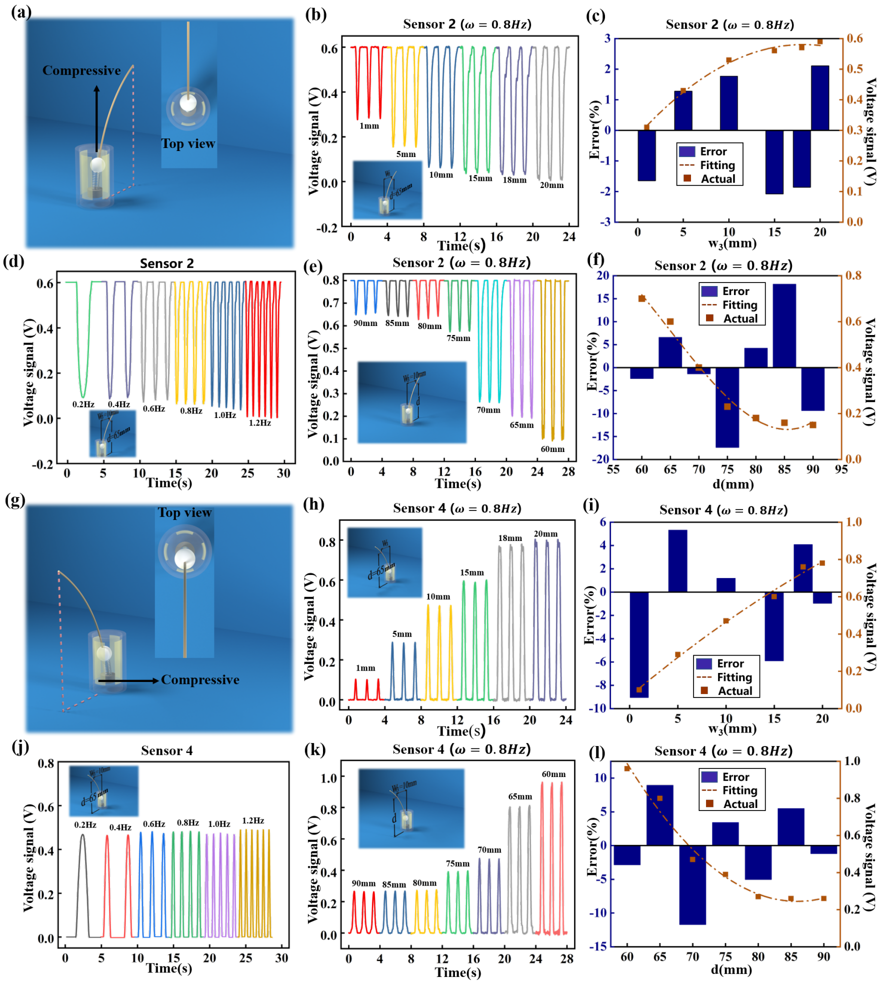

Figure S3: Experimental results. (a) 3DMax model of a whisker and its deformation along the 2 direction starting from a relaxed state. (b) Response by bending to $w_3 = 1 - 20mm$ along the 2 direction. (c) LOOCV validation for evaluating accuracy and generalization ability of 2 regarding w_3. (d) Response from 0.2 to 1.2 Hz in the 2 direction. (e) Response performance at height $d = 60mm - 90mm$ in the 2 direction. (f) LOOCV validation for evaluating accuracy and generalization of 2 regarding d. (g) 3DMax model of whisker and deformation representation along the 4 direction from relaxed state. (h) Response performance by bending $w_3 = 1 - 20mm$ along the 4 direction. (i) LOOCV validation for evaluating accuracy and generalization of 4 regarding w_3. (j) Response from 0.2 to 1.2 Hz along the 4 direction. (k) Response at height $d = 60 - 90mm$ along the 4 direction. (l) LOOCV validation for evaluating accuracy and generalization ability of 4 regarding d.
